# Effects of hyperlipidaemia on plasma apolipoprotein M levels in patients with type 2 diabetes mellitus: an independent case–control study

**DOI:** 10.1186/s12944-016-0325-1

**Published:** 2016-09-15

**Authors:** Puhong Zhang, Jialin Gao, Chun Pu, Gang Feng, Lizhuo Wang, Lizhu Huang, Qingsong Tao, Yao Zhang

**Affiliations:** 1 Department of Biochemistry and Molecular Biology, Wannan Medical College, 22 West Wenchang Road, Wuhu, 241002 People’s Republic of China; 2 Anhui Province Key Laboratory of Biological Macro-molecules Research (Wannan Medical College), Wuhu, China; 3 Department of Clinical Laboratory, Yijishan Hospital of Wannan Medical College, Wuhu, China; 4 Department of Endocrinology and Genetic Metabolism, Yijishan Hospital of Wannan Medical College, Wuhu, China

**Keywords:** Apolipoproteins, Diabetes, HDL, Lipids, Dyslipidaemia

## Abstract

**Background:**

Apolipoprotein M (apoM) is mainly enriched in high-density lipoprotein (HDL) cholesterol and is slightly present in low-density lipoprotein (LDL) cholesterol and very low-density lipoprotein cholesterol. apoM is involved in HDL formation and HDL-mediated reverse cholesterol transport. apoM is also associated with hyperlipidaemia and type 2 diabetes mellitus (T2DM). Significantly high plasma apoM levels are detected in hyperlipidaemia mice with a defective LDL receptor. By contrast, low plasma apoM levels are observed in patients with T2DM, which is often accompanied with hyperlipidaemia. However, the underlying mechanism of this condition is poorly understood. This research aims to examine the changes in apoM levels in patients with hyperlipidaemia and to determine the effects of hyperlipidaemia on plasma apoM levels in patients with T2DM.

**Methods:**

This study included patients with hyperlipidaemia (*n* = 79), patients with T2DM but without hyperlipidaemia (*n* = 125), patients with T2DM and hyperlipidaemia (*n* = 98), and healthy controls (*n* = 105). Their plasma apoM concentrations were measured with enzyme-linked immunosorbent assay.

**Results:**

The average plasma apoM concentrations were 18 % higher in the hyperlipidaemia group (26.63 ± 10.35 ng/μL) than in the healthy controls (22.61 ± 10.81 ng/μL, *P* <0.01). The plasma apoM concentrations were lower in the T2DM without hyperlipidaemia group (18.54 ± 10.33 ng/μL, *P* <0.01) and the T2DM with hyperlipidaemia group (19.83 ± 7.41 ng/μL, *P* <0.05) than in the healthy controls. Similar to apoA-I (1.29 ± 0.33 g/L vs. 1.28 ± 0.31 g/L, *P* >0.05), the plasma apoM concentrations in the T2DM with hyperlipidaemia group did not significantly differ from those in the T2DM without hyperlipidaemia group (*P* >0.05). Multivariate linear regression analysis showed that hyperlipidaemia (β = 5.18, *P* = 0.007) is an independent promoting factor of plasma apoM levels and diabetes (β = −3.09, *P* = 0.005) is an inhibiting factor of plasma apoM levels.

**Conclusion:**

Plasma apoM concentrations are higher in patients with hyperlipidaemia than in healthy controls. Low plasma apoM levels in patients with T2DM are likely caused by diabetes but are not induced by hyperlipidaemia.

## Background

Apolipoprotein M (apoM) is a 25 kDa plasma protein containing 188 amino acids and belonging to the lipocalin protein family [1]. ApoM is highly tissue-specific protein mainly expressed in the liver and kidneys and weakly expressed in the embryonic liver and other tissues [2]. ApoM is the carrier of the biologically active lipid mediator sphingosine-1-phosphate in high-density lipoprotein cholesterol (HDL-C) and is implicated in high-density lipoprotein (HDL) formation and HDL-mediated reverse cholesterol transport [3–5]. Plasma apoM (~23 mg/L) is mainly enriched in HDL-C and is weakly present in low-density lipoprotein cholesterol (LDL-C) and very low-density lipoprotein cholesterol (VLDL-C) [3]. Hyperlipidaemia is often accompanied with complex dyslipidaemia, such as increased LDL-C and VLDL-C levels and low HDL-C levels, which are associated with plasma apoM [6]. Increased plasma triglyceride (TG) levels and significantly high plasma apoM levels are detected in hyperlipidaemia mice with defective low-density lipoprotein (LDL) receptor [7]. This observation suggests that plasma apoM concentrations may change in patients with hyperlipidaemia. The link between apoM and type 2 diabetes mellitus (T2DM) has also been investigated by using in vivo and in vitro models. T2DM has emerged as an increasing threat worldwide, and approximately 15 % of the world’s population will manifest T2DM by 2025 [8]. Plasma apoM levels are increased in streptozotocin-induced diabetic mice but are reduced with insulin treatment [9]. Reduced plasma apoM levels are also observed in another T2DM mouse model with high-fat feed [10]. The latter observation is consistent with a case–control study showing that plasma apoM levels are lower in patients with T2DM than in controls [11]. apoM expression is also altered in HepG2 cells with different concentrations of glucose medium [9, 10]. The apoM promoter variant T-778C is associated with an increased risk of T2DM [12] and is susceptible to type 1 diabetes mellitus [13]. T2DM is often accompanied with hyperlipidaemia, which causes high TG and LDL-C levels and low HDL-C levels [6, 14]. Therefore, the specific effect of hyperlipidaemia on plasma apoM in patients with T2DM should be investigated to confirm whether low plasma apoM levels in T2DM are caused by diabetes or hyperlipidaemia.

This study aimed to examine the apoM levels in patients with hyperlipidaemia, patients with T2DM and hyperlipidaemia, and patients with T2DM but without hyperlipidaemia through enzyme-linked immunosorbent assay (ELISA). This study also aimed to determine the effects of hyperlipidaemia on plasma apoM levels in patients with T2DM.

## Methods

### Subjects

The protocol was approved by the Medical Ethics Committee of the Wannan Medical College in China. All participants (age >18 years) provided written informed consent. Subjects were recruited from The First Affiliated Yijishan Hospital of Wannan Medical College as follows: patients with hyperlipidaemia (*n* = 79), patients with T2DM but without hyperlipidaemia (*n* = 125), patients with T2DM and hyperlipidaemia (*n* = 98), and healthy controls (*n* = 105). All subjects were of Southern Han Chinese ancestry. Healthy control group inclusion criteria include good general health, no significant past medical history, and documented normal fasting blood glucose and glucose intolerance. The exclusion criteria included active inflammatory/infectious disease and history of atherosclerosis, stroke or malignancy. T2DM was defined by the World Health Organization criteria [fasting glucose ≥7.0 mmol/L (126 mg/dL) or 2-h glucose ≥11.1 mmol/L (200 mg/dL)] [15]. The exclusion criteria were non-type 2 diabetes (type 1 diabetes, gestational diabetes, and drug-induced diabetes), pregnancy, thyroid or liver disease, malignancy, azotemia, renal failure, fever, infection, and congestive heart failure. Amongst the diabetic patients, those who used insulin or thiazolidines were also excluded but the use of antihypertensive drugs was allowed [11]. Hyperlipidaemia was defined by the Adult Treatment Panel III of the National Cholesterol Education Program [serum total TG ≥220 mg/dL (2.49 mmol/L) or serum total cholesterol (TC) ≥240 mg/dL (6.24 mmol/L)] [16]. The exclusion criteria were diabetes, pregnancy, thyroid disease, renal or liver disease, malignancy, infection, fever, and congestive heart failure. Hyperlipidaemic patients who used lipid-lowering drugs were also excluded. Blood pressure was measured on the right arm after 15 min rest in a sitting position with a standard mercury sphygmomanometer. All participants had an overnight fast and patients did not take their usual medication and insulin before blood sampling.

### Laboratory analyses

Blood samples were collected in one tube without anticoagulant and one EDTA-containing tube in the morning. We allowed samples without anticoagulant to clot for 2 h at room temperature before centrifugation at ~1000 × *g* for 20 min at 4 °C. The plasma was divided into two tubes; one tube was stored at −80 °C for later assay for apoM, and the other tube with freshly prepared plasma was used for plasma assay (excluding apoM). Whole blood samples in EDTA-containing tubes were gently mixed fully before haemoglobin A1c (HbA1c) assay.

Plasma creatinine (CR), cystatin C (CYS-C), superoxide dismutase (SOD), fasting blood-glucose (FPG), and lipid levels were determined using a Hitachi 7600 biochemistry autoanalyser (Hitachi, Tokyo, Japan). CR, TG, TC, and HDL-C were analysed by enzymatic methods; LDL-C was calculated by the Friedewald formula [17]. CYS-C, apoA-I, apoB, and Lp(a) were analysed by immunoturbidimetric assays; SOD by spectrophotometry; and FPG by glucose oxidase method. All above reagents were purchased from Beijing Leadman Biochemistry Co., Ltd., China. HbA1c was measured by high performance liquid chromatography using a Bio-Rad Variant II analyser (Bio-Rad Laboratories, Hercules, CA, USA), and presented as NGSP units (%) and with International Federation of Clinical Chemistry units (mmol/mol) in parentheses.

Plasma apoM concentrations were assayed using a commercial ELISA kit (Cloud-Clone Corp., Houston, TX, USA) according to the manufacturer’s instructions. The concentrations of apoM in the calibrator were determined using a standard of known apoM concentrations. All samples were diluted at 1:5000 and analysed in duplicate. The range of the standard curve was 0.312–20 ng/mL.

### Statistical analysis

Continuous variables were normally distributed by Kolmogorov–Smirnov test and provided as mean ± SD. Categorical variables were expressed as percentages. Overall comparisons were performed with one-way ANOVA and multiple comparison between the two groups was derived from the LSD-*t* test. Differences in percentages of variables were determined by χ^2^ test. The relationships between the index and apoM were examined using *Pearson* linear regression analysis or multivariable linear regression analysis. Two-sided *P* <0.05 was considered statistically significant. All statistical analyses were performed with SPSS version 16.0.

## Results

### Clinical characteristics and plasma variables including apoM

The plasma apoM concentrations were determined in patients with hyperlipidaemia (*n* = 79), T2DM without hyperlipidaemia (*n* = 125), T2DM with hyperlipidaemia (*n* = 98), and healthy controls (*n* = 105). Comparisons of the baseline characteristics and plasma parameters amongst the four groups are shown in Table 1. No difference was observed in gender and age between the four groups (*P* = 0.673; *P* = 0.347, respectively). Compared with the healthy controls, patients with hyperlipidaemia had higher systolic blood pressure (SBP), diastolic blood pressure (DBP), CR, TC, TG, apoA-I and apoB, and lower HDL-C. SBP, DBP, and plasma FPG levels were higher, whereas SOD, TC, HDL-C and apoA-I levels were lower in the T2DM without hyperlipidaemia group compared with healthy controls. The T2DM with hyperlipidaemia group had higher CYS-C, TC, TG, and apoB levels and lower FPG and HDL-C levels compared with the T2DM without hyperlipidaemia group. SBP, DBP, CYS-C, FPG, and LDL-C levels were higher, whereas SOD, HDL-C, and apoA-I were lower in the T2DM with hyperlipidaemia group compared with those in the hyperlipidaemia group.

**Table 1:** The comparison of clinical characteristics and plasma parameters include apoM

	Healthy controls (*n* =105)	Hyperlipidaemia (*n* = 79)	T2DM without hyperlipidaemia (*n* = 125)	T2DM with hyperlipidaemia (*n* =98)	*P* value
*N* (male/femal)	56/49	45/34	72/53	60/38	0.729
AGE (yrs)	54.65 ± 10.20	53.70 ± 10.61	55.30 ± 12.32	54.82 ± 11.79	0.805
SBP (mmHg)	118.00 ± 12.71	122.94 ± 14.01^*a*^	133.59 ± 18.36^*aaa*^	137.47 ± 19.74^*aaaccc*^	0.000
DBP (mmHg)	76.25 ± 8.37	81.15 ± 10.81^*aa*^	82.74 ± 10.84^*aaa*^	84.86 ± 10.27^*aaac*^	0.000
DM duration (yrs)	-	-	6.00 ± 5.55	6.46 ± 6.25	
HbA1c (%(mmol/mol))	-	-	9.3 ± 2.7(78 ± 30)	9.6 ± 2.0(81 ± 22)	
Cr (umol/L)	63.88 ± 14.76	71.42 ± 14.69^*aa*^	66.88 ± 19.62	70.81 ± 26.75^*a*^	0.026
CYS-C (mg/ L)	0.98 ± 0.22	1.02 ± 0.28	1.00 ± 0.25^*bb*^	1.13 ± 0.39^*aaac*^	0.001
SOD (U/mL)	108.82 ± 10.88	103.85 ± 15.70	81.90 ± 19.01^*aaa*^	88.82 ± 32.17^*aaaccc*^	0.000
FPG (mmol/L)	5.35 ± 0.40	5.32 ± 0.65	8.72 ± 3.09^*aaabbb*^	10.14 ± 3.22^*aaaccc*^	0.000
TC (mmol/L)	4.31 ± 0.57	4.85 ± 1.23^*aa*^	4.00 ± 0.85^*abbb*^	5.00 ± 1.53^*aaa*^	0.000
TG (mmol/L)	1.19 ± 0.45	3.19 ± 0.97^*aaa*^	1.27 ± 0.50^*bbb*^	3.50 ± 1.19^*aaac*^	0.000
HDL-C (mmol/L)	1.43 ± 0.24	1.22 ± 0.22^*aaa*^	1.26 ± 0.34^*aaabbb*^	1.12 ± 0.25^*aaac*^	0.000
LDL-C (mmol/L)	2.33 ± 0.44	2.20 ± 0.90	2.36 ± 0.69^*b*^	2.62 ± 0.93^*aaccc*^	0.002
apoA-I (g/L)	1.66 ± 0.29	1.86 ± 0.31^*aaa*^	1.28 ± 0.31^*aaa*^	1.29 ± 0.33^*aaaccc*^	0.000
apoB (g/L)	0.78 ± 0.22	1.11 ± 0.20^*aaa*^	0.77 ± 0.26^*bbb*^	1.04 ± 0.36^*aaa*^	0.000
LP(a) (mg/L)	124.24 ± 106.99	102.60 ± 109.03	143.13 ± 133.09	126.92 ± 140.27	0.160

Data mean ± SD. *N* number, *M* male, *F* female, *SBP* systolic blood pressure, *DBP* diastolic blood pressure, *HbA1c* glycated hemoglobin A1c, is presented as NGSP units (%) and with IFCC units (mmol/mol) in parentheses., *apo* apolipoprotein; *P value* comparisons among four groups by one-way ANOVA, ^*a*^vs. healthy control group, ^*a*^ *P* <0.05, ^*aa*^ *P* <0.01, ^aaa^ *P* <0.001, ^*b*^vs. T2DM with hyperlipidaemia group, ^*b*^ *P* <0.05, ^*bb*^ *P* <0.01, ^*bbb*^ *P* <0.001, ^*c*^vs. hyperlipidaemia group, ^*c*^ *P* <0.05, ^*cc*^ *P* <0.01, ^*ccc*^ *P* <0.001

**Fig. 1:**
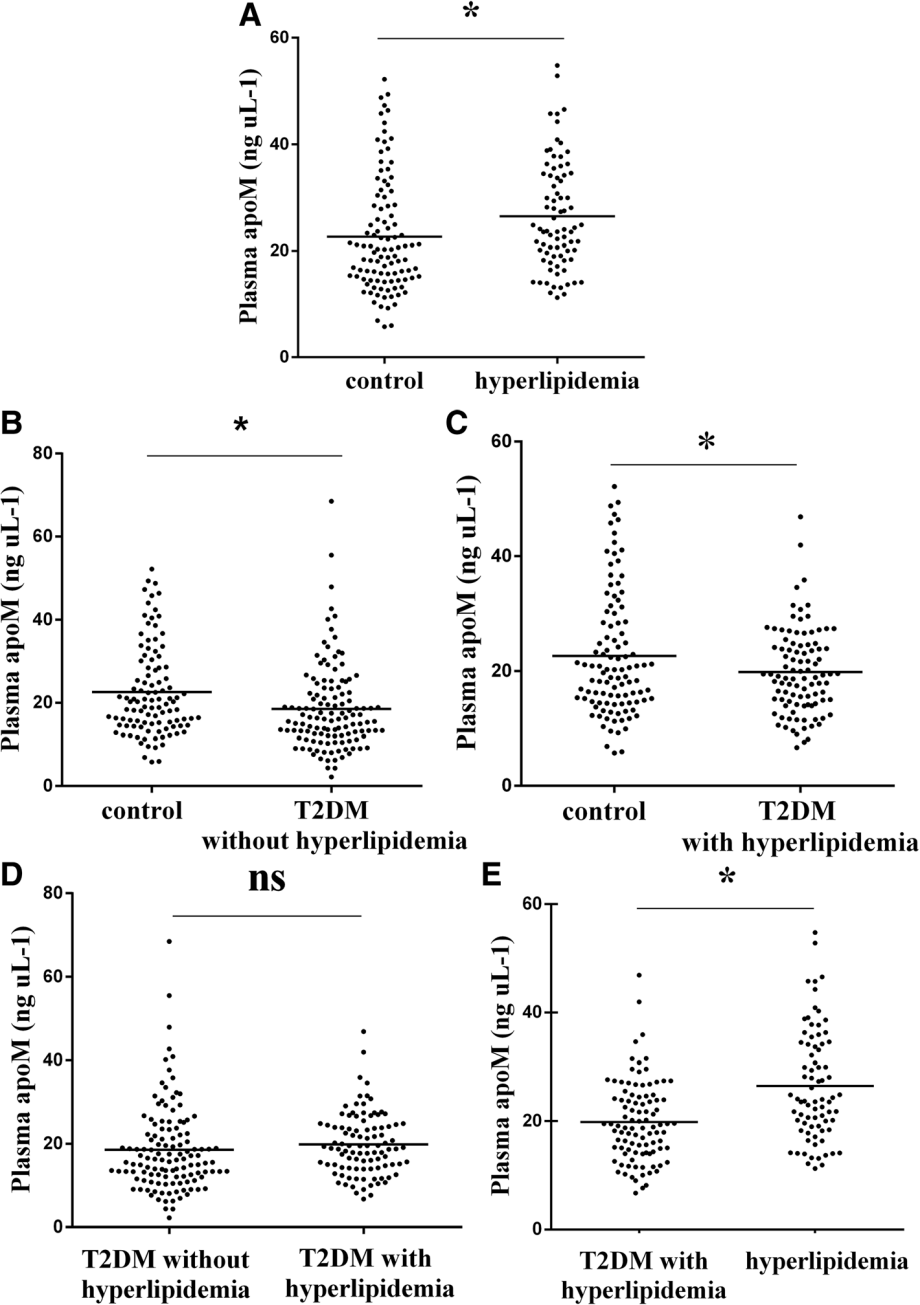
Scatter plot showing the apoM concentrations. hyperlipidaemia(*n* = 79), T2DM without hyperlipidaemia (*n* = 125), T2DM with hyperlipidaemia (*n* = 98), Healthy controls (*n* = 105). **a** only hyperlipidaemia group vs. healthy controls group; (**b**) T2DM without hyperlipidaemia group vs. healthy controls group; (**c**) T2DM with hyperlipidaemia group vs. healthy controls; (**d**) T2DM without hyperlipidaemia group vs. T2DM with hyperlipidaemia group; (**e**) T2DM with hyperlipidaemia group vs. only hyperlipidaemia group. The bars indicate the mean values. *P <0.05

The plasma apoM concentrations were different in four groups (*P* = 0.000, Table 1). The plasma apoM concentrations were on average 18 % higher in hyperlipidaemia patients (26.63 ± 10.35 ng/μL) compared with healthy controls (22.61 ± 10.81 ng/μL; *P* <0.01; Fig. 1a). However, the plasma apoM concentrations were lower in the T2DM without hyperlipidaemia group (18.54 ± 10.33 ng/μL) and T2DM with hyperlipidaemia group (19.83 ± 7.41 ng/μL) compared with those in the healthy control group (22.61 ± 10.81 ng/μL; *P* <0.01, Fig. 1b; *P* <0.05; Fig. 1c, respectively). The plasma apoM concentrations were lower in the T2DM with hyperlipidaemia group (19.83 ± 7.41 ng/μL) compared with those in the hyperlipidaemia group (26.47 ± 10.18 ng/μL, *P* <0.001; Fig. 1e). No statistical difference was observed in plasma apoM concentrations between the T2DM with hyperlipidaemia group (19.83 ± 7.41 ng/μL) and T2DM without hyperlipidaemia group (18.54 ± 10.33 ng/μL, *P* >0.05, Fig. 1d) similar to apoA-I (1.29 ± 0.33 g/L vs. 1.28 ± 0.31 g/L, *P* >0.05, Table 1).

### Association of plasma apoM with clinical and lipid variables

**Fig. 2:**
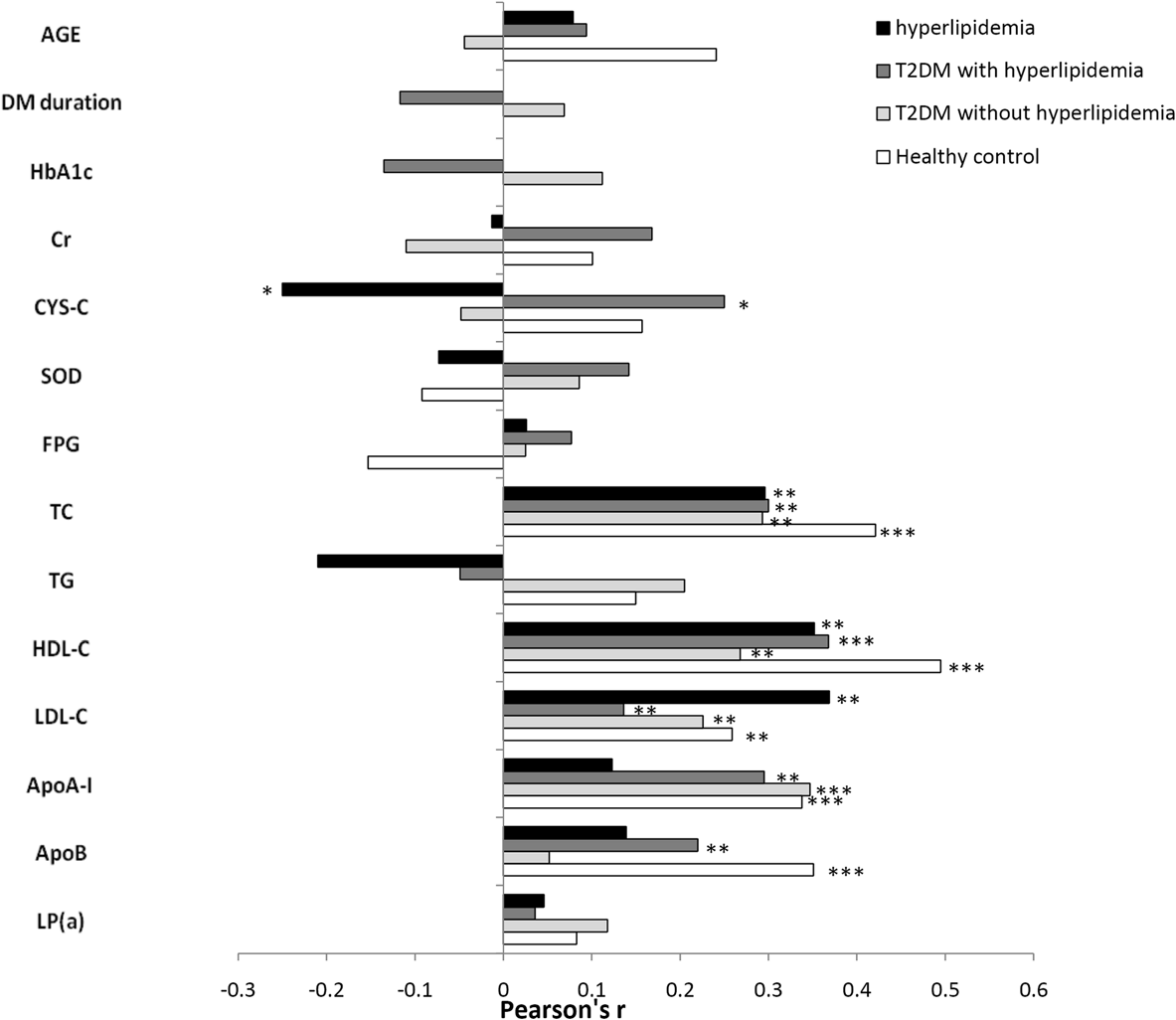
Univariate correlation coefficients (Pearson’s r) for associations of plasma apoM with clinical variables and plasma parameters. hyperlipidaemia (*n* = 79), T2DM without hyperlipidaemia (*n* = 125), T2DM with hyperlipidaemia (*n* = 98), and healthy controls (*n* = 105). The bars indicate the mean values. *P <0.05;**P <0.01; ***P <0.001

The plasma apoM concentration was negatively correlated with CYS-C in patients with hyperlipidaemia alone (*r* = −0.250, *P* <0.05). However, apoM was positively correlated with CYS-C in the T2DM with hyperlipidaemia group (*r* = 0.250, *P* <0.05) but was not correlated with CYS-C in the other two groups. Amongst the lipid-related variables, apoM was positively correlated with TC, HDL-C, and LDL-C in the hyperlipidaemia group (*r* = 0.296, *P* <0.01; *r* = 0.352, *P* <0.01; and *r* = 0.369, *P* <0.01, respectively), T2DM with hyperlipidaemia group (*r* = 0.300, *P* <0.01; *r* = 0.368, *P* <0.001; and *r* = 0.136, *P* >0.05, respectively), T2DM without hyperlipidaemia group (*r* = 0.293, *P* <0.01; *r* = 0.268, *P* <0.01; and *r* = 0.226, *P* <0.05), and in healthy controls (*r* = 0.421, *P* <0.001; *r* = 0.495, *P* <0.001; and *r* = 0.259, *P* <0.001, respectively). Besides that, apoM was also positively correlated with apoA-I in the T2DM with hyperlipidaemia group (*r* = 0.030, *P* <0.01), T2DM without hyperlipidaemia group (*r* = 0.347, *P* <0.001), and healthy controls (*r* = 0.338, *P* <0.001), although such association was not found in the hyperlipidaemia group (*r* = 0.123, *P* >0.05). ApoM was also positively correlated with apoB in the T2DM with hyperlipidaemia group (*r* = 0.220, *P* <0.05) and healthy controls (*r* = 0.351, *P* <0.001). However, this association was not found in the hyperlipidaemia alone group (*r* = 0.139, *P* >0.05) and T2DM without hyperlipidaemia group (*r* = 0.052, *P* >0.05). ApoM was not correlated with age, diabetes mellitus(DM) duration, ACR, FPG, HbA1c, CR, SOD, TG, and LP(a) in the four groups (Fig. 2). By multivariate linear regression analysis, TC (β = 1.33, *P* = 0.007), HDL-C (β = 5.16, *P* = 0.016), and apoA1 (β = 5.18, *P* = 0.007) were the independently influential factors for plasma apoM concentrations (Table 2). Meanwhile, T2DM itself (β = −3.09, *P* = 0.005) was the independently negative factor for apoM, whereas hyperlipidaemia (β = 3.43, *P* = 0.001) was the positive factor. Moreover, no interaction was observed between each other (Table 3).

**Table 2:** Influential factors for apoM:multivariable linear regression analysis

	β	SE	t	*P* value	95 % Confidence Interval for β	
TC	1.33	0.49	2.70	0.007	0.36	2.29
HDL	5.16	2.14	2.41	0.016	0.94	9.38
apoA1	5.18	1.93	2.69	0.007	1.39	8.97

*β* unstandardized coefficients, *SE* standardized error, *β’* standardized coefficients

**Table 3:** T2DM and hyperlipidaemia are independently influential factor for apoM

	β	SE	t	*P* value	95 % Confidence Interval for β	
T2DM	−3.09	1.09	−2.83	0.005	−5.23	−0.94
hyperlipidemia	3.43	1.01	3.40	0.001	1.44	5.41

*β* unstandardized coefficients, *SE* standardized error, *β’* standardized coefficients

## Discussion

Our study mainly revealed that plasma apoM concentrations in patients with hyperlipidaemia were higher than those in healthy controls. Hyperlipidaemia was an independent promoting factor of plasma apoM levels. Diabetes was also an independent inhibiting factor of plasma apoM levels. These observations suggested that hyperlipidaemia increased plasma apoM levels. Therefore, low plasma apoM levels in T2DM patients were not caused by hyperlipidaemia. Instead, low plasma apoM levels were likely triggered by diabetes.

ApoM accounts for ~5 % of HDL-C and <2 % of LDL and was one of the main components of HDL-C. The apoM complex also contains HDL-C (apoA-I)- and LDL-C (apoB)-related apolipoproteins [3]. Thus, plasma apoM concentrations (~23 mg/L) were associated with HDL-C, and also showed a strong association with LDL-C, TC, apoA-I, and apoB [11, 18, 19]. This study also confirmed the relationship between plasma apoM and HDL-C, LDL-C, apoA-I, and apoB by univariate linear regression analysis. In addition, apoM was independently associated with HDL-C, TC, and apoA1 by multivariate analyses. Hyperlipidaemia often displays reduced plasma HDL-C and increased TC and LDL-C, which was associated with apoM [6, 14]. This study determined the plasma apoM levels in patients with hyperlipidaemia and found that hyperlipidaemia patients have marked apoM levels than healthy controls. Moreover, hyperlipidaemia was an independently promoting factor for plasma apoM. Both the plasma apoA-I and apoM levels were higher than those in healthy controls. Besides that, both the plasma apoA-I and apoM levels were not statistically different between the T2DM without hyperlipidaemia groups and T2DM with hyperlipidaemia groups. Multivariate linear regression analysis also confirmed that the plasma apoA1 level was the independent influential factor for apoM. Faber et al. had found that the plasma concentrations of apoM decreased to 33 % of the normal value in mice deficient in apoA-I [20]. In general, these observations showed that the synthesis, secretion or turnover of apoM in plasma may to a certain extent depend on apoA-I. The fractional catabolic rate of apoA-I is significantly increased, whereas the absolute production of apoA-I is inhibited in T2DM patients and thus causes a decrease in plasma apoA-I levels [21–23]. Meanwhile, apoA-I and apoM mainly exist in HDL-C [24], and a connection is observed between apoM and apoA-I metabolism [20]. This condition suggests that the the metabolism of apoM in T2DM may be partly similar to apoA-I.

In two independent case–control studies, the mean HDL-C level is significantly lower in coronary heart disease patients than in control subjects, but the mean apoM levels do not significantly differ between the two groups [25]. Similarly, the apoM levels in T2DM with hyperlipidaemia patients also showed no significant difference compared with patients of T2DM without hyperlipidaemia, whereas the HDL-C level was significantly lower in the former, suggesting that plasma apoM levels were also correlated with other factors, such as LDL-C, TC, and apoA-I, except for HDL-C [11, 18, 26]. These results agree with data from Christoffersen et al., which indicates that the mice with defective LDL receptor whose plasma TG levels were increased had significantly high plasma apoM and the large fraction of apoM was associated with LDL-C/CM particles and not HDL-C [7]. Meanwhile, Kurano observed that in LDL receptor-overexpressing mice, the plasma apoM levels were decreased [27]. Karuna et al. also found that apoM is mainly present in plasma LDL-C and has a negative correlation with HDL-C concentration in individuals with low HDL-C because of lecithin–cholesterol acyltransferase deficiency [28]. A possible explanation for the above results and the relationship between hyperlipidaemia and plasma apoM levels could be that apoM can rapidly exchange between HDL-C and VLDL-C/LDL-C particles in vivo [7]. This condition leads to the VLDL/LDL pool of apoM that is replenished from the HDL in hyperlipidaemia patients with marked plasma VLDL/LDL levels.

Hepatic apoM gene expression and plasma level were significantly low in mouse models of alloxan-induced diabetes and hyperglycaemia-induced diabetes [29, 30]. Meanwhile, the plasma apoM concentration was lower in patients with T2DM compared with those in healthy controls [31]. This study confirmed that the plasma apoM levels were reduced in T2DM patients with or without hyperlipidaemia than those in healthy controls. Multivariate analyses showed that diabetes itself was an independently inhibiting factor for plasma apoM levels. However, the specific reasons are still unclear. Previous study reported that insulin and high glucose (30 mmol/L glucose) can downregulate apoM expression in HepG2 cell culture and diabetic mouse [26]. However, a recent research found that the influences of 13.5 g/dL glucose, 9 and 4.5 g/dL glucose on apoM in HepG2 cell were not statistically different. Whereas, the medium apoM and cellular apoM levels significantly increased accompanied with elevator glucose (0, 1.0, and 4.5 g/dL). Besides, the plasma apoM levels were higher in streptozotocin-induced diabetes mice than those in control mice [9]. Hyperglycaemia can stimulate the hexosamine pathway and cause an increase in endogenous glucosamine levels [32], but both exogenous and endogenous glucosamines can increase the apoM expression in HepG2 cells and in rat models [33]. Our study did not observe a correlation between plasma apoM and glucose/HbA1c in healthy subjects or T2DM patients similar to that described in a previous report [31]. Previous studies indicated that the effects of blood glucose concentrations on apoM level are complex. Glucose concentrations, glucose metabolic products, and diabetes pathogenesis can influence apoM metabolism.

Family history of diabetes and pre-diabetic condition can be accounted for an increase in the risk of insulin resistance and high blood glucose concentrations, which can induce endothelial impairment and exacerbate dyslipidaemia and atherosclerotic lesions [34]. Interestingly, nutraceuticals and functional food ingredients, such theaflavins and proanthocyanidins, which elicit lipid-lowering effects, may also influence plasma apolipoprotein levels [35].

## Conclusions

In conclusion, plasma apoM concentrations were higher in patients with hyperlipidaemia than in healthy subjects. Hyperlipidaemia was an independent promoting factor of plasma apoM levels. Diabetes was also an independent inhibiting factor of plasma apoM levels. Therefore, low plasma apoM levels in T2DM patients were likely caused by diabetes but were unlikely induced by hyperlipidaemia.

## Abbreviations

apo: Apolipoprotein
Cr: Creatinine
CYS-C: Cystatin C
DBP: Diastolic blood pressure
ELISA: Enzyme linked immunosorbent assay
FPG: Fasting blood-glucose
HbA1c: Hemoglobin A1c
HDL-C: High-density lipoprotein cholesterol
LDL-C: Low-density lipoprotein cholesterol
Lp(a): Lipoprotein (a)
SBP: Systolic blood pressure
SOD: Superoxide dismutase
T2DM: Type 2 diabetes mellitus
TC: Total cholesterol
TG: Triglyceride
VLDL: Very low-density lipoprotein cholesterol
